# Robust control of heart rate for cycle ergometer exercise

**DOI:** 10.1007/s11517-019-02034-6

**Published:** 2019-08-30

**Authors:** Kenneth J. Hunt, Cédric C. Hurni

**Affiliations:** Institute for Rehabilitation and Performance Technology, Division of Mechanical Engineering, Department of Engineering and Information Technology, Bern University of Applied Sciences, Burgdorf, CH-3400 Switzerland

**Keywords:** Heart rate control, Heart rate dynamics, Heart rate variability, System identification, Physiological control, Cycle ergometers

## Abstract

The objective was to assess the performance and robustness of a novel strategy for automatic control of heart rate (HR) during cycle ergometry. Control design used a linear plant model and direct shaping of the closed-loop input-sensitivity function to achieve an appropriate response to disturbances attributable to broad-spectrum heart rate variability (HRV). The controller was evaluated in 73 feedback control experiments involving 49 participants. Performance and stability robustness were analysed using a separately identified family of 73 plant models. The controller gave highly accurate and stable HR tracking performance with mean root-mean-square tracking error between 2.5 beats/min (bpm) and 3.1 bpm, and with low average control signal power. Although plant parameters varied over a very wide range, key closed-loop transfer functions remained invariant to plant uncertainty in important frequency bands, while infinite gain margins and large phase margins (> 62^∘^) were preserved across the whole plant model family. Highly accurate, stable and robust HR control can be achieved using LTI controllers of remarkably simple structure. The results highlight that HR control design must focus on disturbances caused by HRV. The input-sensitivity approach evaluated in this work provides a transparent method of addressing this challenge.

**Graphical Abstract:**
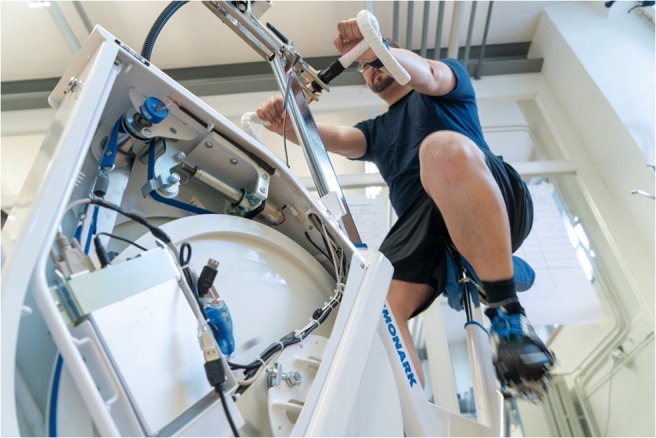
Heart rate control using a cycle ergometer

Heart rate control using a cycle ergometer

## Introduction

Well-established guidelines exist for the definition of cardiopulmonary exercise testing protocols and for the prescription of training regimes. Specific testing and prescription guidelines are available for healthy individuals and for patients across a diversity of health conditions [1]; the most common exercise modalities are treadmill walking/running and cycle ergometry, while exercise intensity can be characterised using such variables as heart rate (HR), oxygen uptake or a subjective rating of perceived exertion, RPE [2].

HR is a quantitative variable that can be easily measured, and several approaches have been investigated for automatic control of HR during both treadmill and cycle ergometer exercise ([3–5] and [6–8], respectively). These feedback systems facilitate tracking of arbitrary HR profiles by automatically and continuously adjusting a manipulated variable, which for treadmills can be speed or slope, or both, and which for cycling is usually work rate.

Since cycle ergometers provide a stable-seated position, they are the preferred modality for exercise testing and prescription in cardiac rehabilitation; HR controllers have long been investigated in this context [6, 9, 10], but also, subsequently, for healthy persons [7, 8, 11].

A most elegant treatment of HR control for cycle ergometers was provided by Kawada et al. [6]. In that work, a single linear transfer function model of HR response to changes in work rate was obtained as an average from open-loop system identification experiments with 10 individual participants (8 men, 2 women). The model was then used in simulation to tune the two free parameters of a linear proportional-integral (PI) controller. The single time-invariant PI controller thus obtained was then tested in HR control experiments with 55 healthy participants (45 men, 10 women) and with 12 patients with cardiac disease (10 men, 2 women). In the healthy participants (*n* = 55), the mean root-mean-square tracking error (RMSE) for a constant HR target of 60 % of maximal HR (HRmax) was 2.5 beats/min (bpm); when the HR target was 75 % of HRmax, mean RMSE was 3.8 bpm. For the cardiac patients (*n* = 12) exercising at a constant target HR of 20 bpm above resting HR, mean RMSE was 3.0 bpm. This work, which reported HR control data from 122 individual HR control tests with 67 participants in the two experimental cohorts, thus provides strong empirical evidence that a single linear, time-invariant (LTI) controller of very simple structure can provide accurate and robust HR control.

A variety of nonlinear approaches to HR modelling and control for both treadmills and cycle ergometers have been proposed. Nonlinear models have been used to represent the different gains and time constants that exist for positive and negative step changes in speed [12]; asymmetry has also been observed and modelled during moderate-intensity treadmill running [13]. For the purpose of control design, a nonlinear state-space model, where the control signal appears in quadratic form, was employed and combined with linear-quadratic and H-infinity optimisation [14]; the same model structure was used, but with a nonlinearity-cancellation strategy, for HR control using a treadmill [4] or cycle ergometer [7]. A related approach using a Hammerstein model structure and a compensator with cancellation of the nonlinear model term was combined with model-predictive control [15]. Other approaches include linear H-infinity control with static nonlinearity compensation [16] and a nonlinear neural network approach [17]. A limitation common to most of these reports is that quantitative measures of controller performance (i.e. RMSE and control signal intensity) were not employed and that very small numbers of participants were included in experimental evaluations, thus making it difficult to objectively gauge their utility.

A recent study of HR control during cycle ergometry combined an LTI proportional-integral-derivative (PID) controller with an auditory biofeedback signal [8]. Despite the human-in-the-loop nature of this approach, quite accurate tracking was achieved with mean RMSE on the range 3.7 bpm to 5.0 bpm (various experiments with 24 healthy male participants).

In concordance with some of the above observations, a growing body of evidence has emerged from treadmill studies that points towards heart rate variability (HRV, [18]) as the principal challenge in the design of HR control systems, in contradistinction to parametric and/or structural sources of plant uncertainty. From a control-theoretical perspective, HRV presents as a broad-spectrum disturbance signal [19]; care must therefore be taken to ensure that the control signal is not unduly excited at frequencies that might disturb the exercising subject. In short, the said studies have demonstrated that simple approximate linear models, [20], can be employed to design LTI controllers that give highly accurate, stable and robust HR control performance, e.g. [5, 21] (20 to 30 participants, mean RMSE below 3 bpm).

To directly address the HRV disturbance, a HR control approach was developed that allows the frequency-domain characteristics of the closed-loop input-sensitivity function, which is the transfer function from the HRV disturbance to the control signal, to be appropriately shaped [5]; for treadmill exercise, HR control was accurate (mean RMSE of 3.0 bpm, *n* = 30) and the control signal was smooth and stable (average power of changes in the control signal was low). Using this design approach as a foundation, and based on the observation that HR dynamics are not significantly different between treadmills and cycle ergometers, [22], a common control strategy was derived and experimentally tested with these two exercise modalities; it was found to give accurate tracking (mean RMSE of 3.1 bpm vs. 2.8 bpm, cycle ergometer vs. treadmill; *n* = 25) and low control signal intensity [11].

The primary contribution of the present work is, for the first time, the application of the input-sensitivity-shaping approach for feedback control of HR to cycle ergometer exercise and the systematic analysis of its performance and robustness in a large experimental test series. A secondary contribution is a comparison with alternative linear and nonlinear controllers based upon data available in the literature. A single LTI feedback compensator was calculated using a linear first-order plant model. The aim of the work was to assess controller performance in several experimental scenarios using quantitative measures of tracking accuracy and control signal intensity (a total of 73 feedback control experiments involving 49 individual participants were performed), and to analyse performance and stability robustness properties of the compensator using a large family of empirically derived plant models (73 individual plant models were used for the robustness analysis).

## Methods

### Control design

**Fig. 1:**
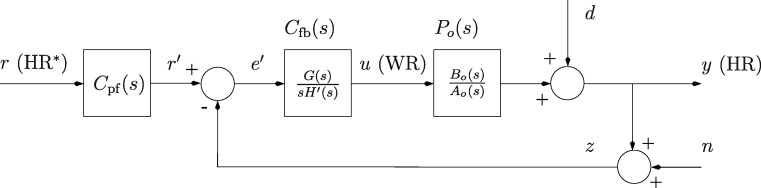
Control structure. *y* is the controlled variable (heart rate [HR]), *u* is the control signal (target work rate [WR]) and *r* is the target heart rate (HR^∗^). The disturbance *d* includes physiological heart rate variability, and *n* is a notional measurement noise signal

The HR control system includes the plant (the nominal plant is denoted *P*_*o*_ and off-nominal plants as *P*), a feedback compensator *C*_fb_ and a reference prefilter *C*_pf_ (Fig. 1). The signals which are present in the generic control structure are interpreted as follows: the controlled variable (plant output) *y* represents HR; the control signal (plant input) *u* is the target work rate (WR) that is computed continuously by the compensator; *r* is the target HR (HR^∗^); the output disturbance *d* represents physiological heart rate variability and other sources of uncertainty; *n* is a notional measurement noise signal; *z* is the HR measurement; and $r^{\prime }$ and $e^{\prime }$ are intermediate signals.

The nominal plant *P*_*o*_ that was used for controller calculation is the strictly proper transfer function as follows:

1 $$ u \rightarrow y :  P_{o}(s) = \frac{B_{o}(s)}{A_{o}(s)} = \frac{k}{\tau s + 1} = \frac{0.392}{65.6 s + 1}, $$

where the specific values for steady-state gain (*k* = 0.392 bpm/W) and time constant (*τ* = 65.6 s) are averages taken over 25 individual participants from a previous system identification study [22].

The lumped linear plant model, Eq. (1), is theoretically valid for small-signal deviations around a nominal operating point; under this approximating condition, the HRV disturbance term *d* acts as an additive output disturbance independent of the control signal *u*. Furthermore, it is recognised that different levels of exercise intensity, as characterised by HR, will lead to different levels of HRV [19].

The feedback compensator *C*_fb_ is a transfer function that is required to be strictly proper and includes an integrator as follows:

2 $$ e^{\prime} \rightarrow u :  C_{\text{fb}}(s) = \frac{G(s)}{H(s)} = \frac{G(s)}{sH^{\prime}(s)};  n_{g} < n_{h}, $$

where *n*_*g*_ and *n*_*h*_ are the degrees of polynomials *G* and *H*, respectively.

The reason for the strictly proper constraint on *C*_fb_ is that the resulting input-sensitivity function *U*_*o*_, Eq. (6) below, is also strictly proper as follows: as a consequence, both *C*_fb_ and *U*_*o*_ are low pass systems (i.e. HCode $\lim _{\omega \rightarrow \infty } |C_{\text {fb}}(j\omega )| = 0$ and $\lim _{\omega \rightarrow \infty } |U_{o}(j\omega )| = 0$), thus making the feedback loop, and, in particular, the control signal *u*, insensitive to high-frequency noise and disturbances.

The compensator was structured in such a way that the closed-loop input-sensitivity function is of first order with a given bandwidth *p*. This is achieved as documented in [5] using the following:

3 $$ C_{\text{fb}}(s) = \frac{\frac{p}{k} (s + \frac{1}{\tau})}{s(s + p + \frac{1}{\tau})} $$

wherefore *C*_fb_ depends only on the design parameter *p*, and on the given plant gain *k* and time constant *τ*. The input-sensitivity bandwidth was chosen to be 0.01 Hz, thus *p* = 0.0628 rad/s and, with *k* = 0.392 and *τ* = 65.6,

4 $$ C_{\text{fb}}(s) = \frac{0.160 s + 0.00244}{s(s + 0.0781)}. $$

The reference prefilter *C*_pf_ was calculated to make the overall closed-loop transfer function from reference *r* to output *y* equal to a standard second-order system with critical damping and specified rise time ([5, 11]; here, the rise time was set to either 120 s [control tests with cohort A, see Section 2.4] or 150 s [cohort B]).

Using the above structures and parameters for *P*_*o*_ and *C*_fb_, the nominal loop gain *L*_*o*_ is obtained as follows:

5 $$ \begin{array}{@{}rcl@{}} L_{o}(s) &=& C_{\text{fb}}(s)P_{o}(s) = \frac{p(s + \frac{1}{\tau})}{s(s + p + \frac{1}{\tau})(\tau s + 1)}\\ &=& \frac{0.0628s + 0.000957}{s(65.6s^{2} + 6.12s + 0.0781)}, \end{array} $$

which gives an infinite gain margin and a phase margin of 81.2^∘^ (Nyquist plot, Fig. 8).

Similarly, the nominal closed-loop input sensitivity (*U*_*o*_), sensitivity (*S*_*o*_) and complementary sensitivity (*T*_*o*_) functions are as follows:

6 $$  d, r^{\prime}, n \rightarrow u :  U_{o}(s) = \frac{C_{\text{fb}}(s)}{1 + L_{o}(s)} = \frac{\frac{p}{k}}{s + p} = \frac{0.160}{s + 0.0628}, $$

7 $$ \begin{array}{@{}rcl@{}} d \rightarrow y :  S_{o}(s) &=& \frac{1}{1 + L_{o}(s)} = \frac{s(s + p + \frac{1}{\tau})}{(s + p)(s + \frac{1}{\tau})}\\ &=& \frac{s(s + 0.0781)}{s^{2} + 0.0781s + 0.000957}, \end{array} $$

and

8 $$ \begin{array}{@{}rcl@{}} r^{\prime}, n \rightarrow y :  T_{o}(s) &=& \frac{L_{o}(s)}{1 + L_{o}(s)} = \frac{\frac{p}{\tau}}{(s + p)(s + \frac{1}{\tau})}\\ &=& \frac{0.000957}{s^{2} + 0.0781s + 0.000957}. \end{array} $$

**Fig. 2:**
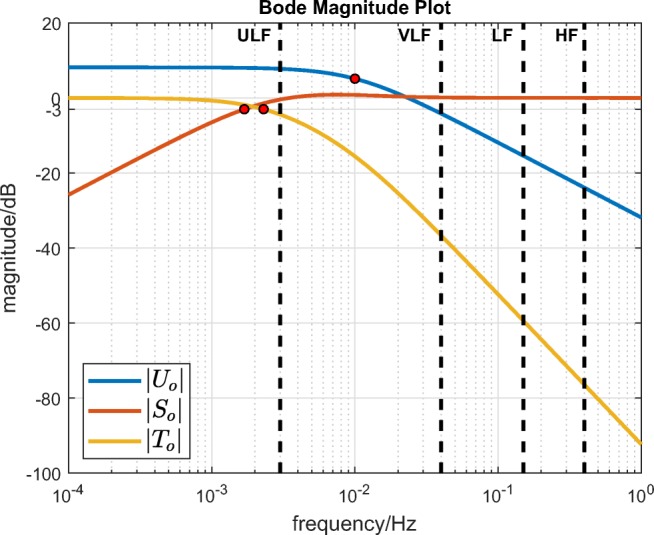
Nominal frequency responses: input sensitivity |*U*_*o*_|, sensitivity |*S*_*o*_| and complementary sensitivity |*T*_*o*_|. The red dots mark the − 3 dB bandwidths. The frequency bands used for analysis of heart rate variability are delineated by dashed vertical lines (ULF, ultra low frequency; VLF, very low frequency; LF, low frequency; HF, high frequency; [18])

The magnitudes of the nominal frequency responses are displayed in a Bode plot (Fig. 2).

From Eq. 6, it is seen that the effect of the HR disturbance term *d* on the controller output signal *u* is governed by the input-sensitivity function *U*_*o*_, which effectively acts as a filter for *d*. Thus, explicit shaping of the frequency response of *U*_*o*_ obviates the need for any separate filtering of the HR signal.

### Materials

**Fig. 3:**
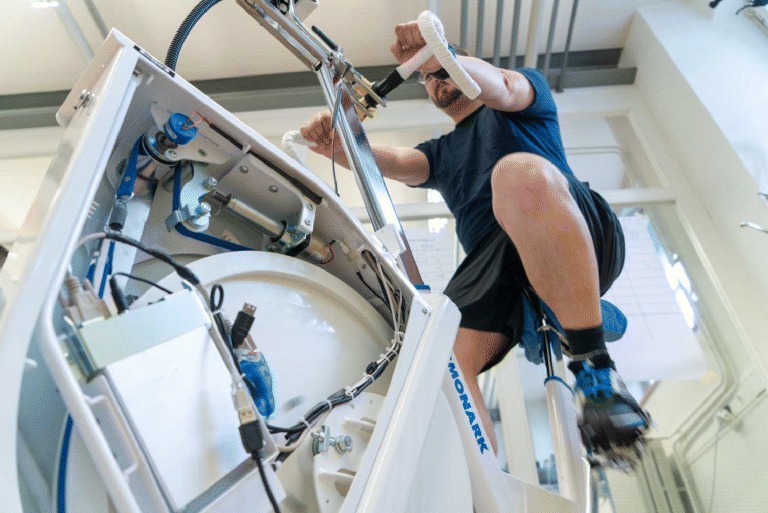
Computer-controlled cycle ergometer (Monark LC7)

All tests were carried out with a commercial cycle ergometer (LC7 by Monark Exercise AB, Sweden; Fig. 3), connected via USB serial link to a PC and controlled in real time using Simulink (The Mathworks, Inc., USA). The control signal *u*, computed as the output of the compensator transfer function *C*_fb_, is a target work rate that is sent to the cycle ergometer; the cycle has an on-board microcontroller and firmware with a feedback controller that continuously adjusts the flywheel load in order to meet the target work rate.

HR was recorded using a chest belt (T34, Polar Electro Oy, Finland) that communicated wirelessly with a receiver module (Heart Rate Monitor Interface [HRMI], Sparkfun Electronics, USA) connected via USB to the PC. The HR signal was interfaced to the Simulink model using a sample rate of 1 Hz. The feedback controller ran at a rate of 0.2 Hz (sample interval of 5 s); at each controller sample instant, the current HR value was taken as the mean of the latest five discrete HR samples.

The choice of controller sample interval of 5 s was based on formal guidelines for closed-loop control systems: it is recommended that the controller sampling rate should be 10 to 30 times the closed-loop bandwidth [23, page 110]. Since the chosen bandwidth for the closed-loop input-sensitivity function is 0.01 Hz, the appropriate range for sampling frequency is 0.1 Hz to 0.3 Hz. Here, the sampling rate was chosen to be exactly in the middle of this range, viz. 0.2 Hz, corresponding to a sample interval of 5 s. Since the raw HR signal was available at a rate of 1 Hz, it was resampled to the appropriate controller sampling rate of 0.2 Hz as described above. The dynamic effect of this resampling is considered to be negligible, given that the plant time constant of 65.6 s is an order of magnitude higher than the controller sample interval of 5 s. Furthermore, the sampling rate of 0.2 Hz is more than a decade above the chosen closed-loop input-sensitivity bandwidth of 0.01 Hz; this choice of sampling rate will therefore lead to no appreciable effects of the HR resampling on the closed-loop system.

### Outcome measures

To obtain a quantitative measure of the accuracy of HR tracking, the RMSE between the nominal and measured HR values (HR_nom_ and HR, respectively) was computed as follows:

9 $$  \text{RMSE} = \sqrt{\frac{1}{N}\sum\limits_{i=1}^{N}(\text{HR}_{\text{nom}}(i) - \text{HR}(i))^{2}}, $$

which has the same units as HR itself, viz. bpm. Here, *i* are the discrete time indices for the 5-s controller sampling interval. HR_nom_ was obtained by simulation of the nominal closed-loop reference response.

Control signal intensity was numerically quantified using the average power of sample-to-sample changes in the control signal *u*. This average control signal power outcome, denoted *P*_∇*u*_, is defined as follows:

10 $$  P_{\nabla u} = \frac{1}{N-1}\sum\limits_{i=2}^{N}(u(i) - u(i-1))^{2}. $$

Since the control signal is the target work rate, the units of *P*_∇*u*_ are W^2^.

For tests involving a square-wave HR target profile, RMSE and *P*_∇*u*_ were calculated over an evaluation period from 300 to 1800 s; when the target HR was constant, the evaluation period was from 400 to 1200 s.

### Experimental procedures

System identification and feedback control experiments were carried out with two separate sets of participants, referred to in the sequel as cohorts A and B:

- Participant cohort A comprised 25 males (*n* = 25) with age on the range 22 years to 32 years, body mass from 62 to 114 kg, height from 1.65 to 1.93 m and body mass index (BMI) from 19.9 to 34.0 kg/m^2^. This cohort previously participated in separate studies of system identification [22] and feedback control [11] using both a cycle ergometer and a treadmill. As noted above (Section 2.1), the nominal plant parameters *k* and *τ* in Eq. 1 that were used for controller calculation are average values obtained in the identification experiments with cohort A [22].
- Participant cohort B had 24 males (*n* = 24) aged from 22 to 36 years, mass from 62 to 113 kg, height from 1.72 to 2.00 m and BMI from 18.8 to 32.5 kg/m^2^. Two system identification series were conducted with cohort B: the first series (denoted B1) used the same experimental protocol applied to cohort A, [22], with a constant cycling cadence of 70 rpm; in the second series (B2), participants were allowed to cycle at their own preferred cadence, which was allowed to vary. During these tests, ratings of perceived exertion (RPE) were recorded four times at intervals of five minutes using the Borg RPE scale [2].

The 73 plant models so identified (results in Section 3.2) were used to analyse performance and stability robustness of the feedback compensator *C*_fb_ (Section 3.3).

Three sets of feedback control experiments were carried out, one with cohort A and two with cohort B:

- Cohort A (*n* = 25): a square-wave target HR profile was employed with a period of 10 min and variations of ± 10 bpm around an individual, moderate-to-vigorous intensity HR level denoted HRmid and calculated as described below. Participants maintained a constant cadence of 70 rpm by monitoring a visual display.
- Cohort B (*n* = 24): the target HR was constant and equal to HRmid; cadence was constant at 70 rpm.
- Cohort B (*n* = 24): target HR was again constant at HRmid, but participants were able to self-select their preferred cadence.
  For cohort B, the order of presentation of the two test conditions (constant or freely chosen cadence) was counterbalanced by changing the test order for consecutive participants.

Thus, a total of 73 feedback control tests involving 49 individual participants were conducted using the single feedback compensator *C*_fb_, Eq. 4.

The mid-level, moderate-to-vigorous intensity HRmid used to design the target HR profiles was calculated individually for each participant as follows. The boundary between exercise intensities perceived to be moderate and vigorous occurs at 76.5 % of maximal HR [1], and maximal HR can be estimated as HRmax (bpm) = 220 − age (years) [24]. Furthermore, it has been documented that, for similar levels of perceived exercise intensity, HR on a cycle ergometer is approximately 20 bpm lower than on a treadmill [25]. Thus, the mid-level HR target was set to HRmid (bpm) = 0.765 × (220 − age) − 20.

## Results

### Feedback control

**Fig. 4:**
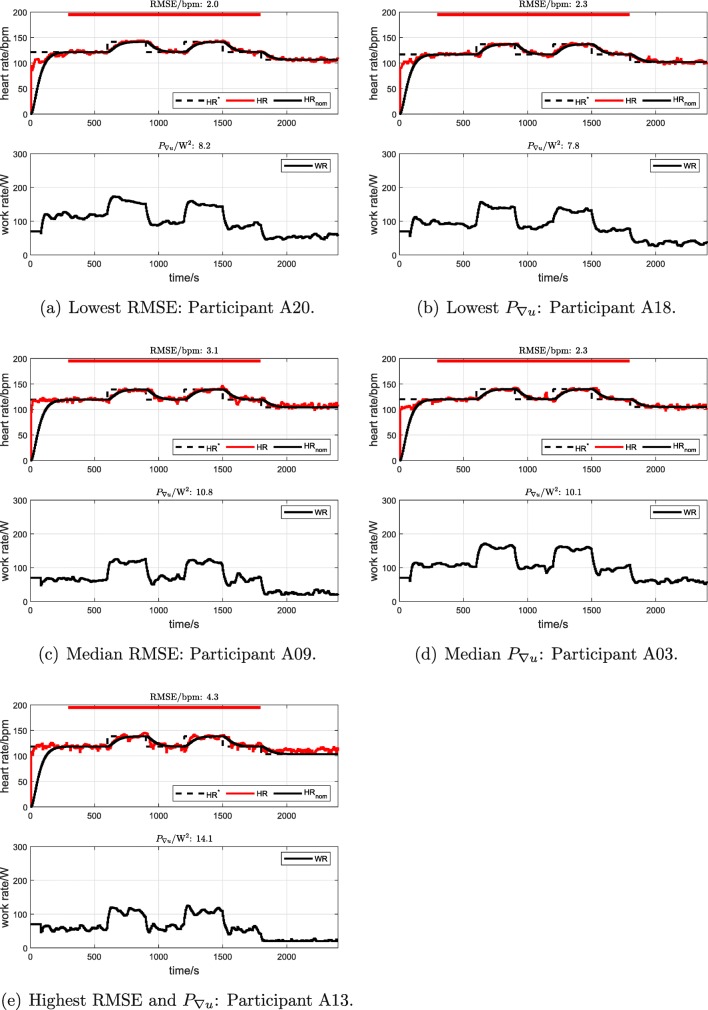
Participant cohort A: individual HR control tests with the minimum, median and maximum values of RMSE (parts a, c and e) and of *P*_∇*u*_ (parts b, d and e). “A20” refers to cohort A, participant number 20, etc. The red bars depict the outcome-evaluation interval from 300 to 1800 s. (Parts a, c and e adapted from [11] to express *P*_∇*u*_ in absolute terms (units of W^2^), instead of after normalisation [units of bpm^2^])

For participant cohort A (*n* = 25, cadence = 70 rpm), tested using a square-wave HR target profile, RMSE was 3.10 bpm ± 0.68 bpm (mean ± standard deviation) with a range of 1.99 bpm to 4.29 bpm. The average control signal power *P*_∇*u*_ was 10.34 W^2^ ± 1.73 W^2^ (range 7.84 W^2^ to 14.07 W^2^). Original data records for the individual cohort A tests with the minimum, median and maximum values of RMSE and of *P*_∇*u*_ are shown in Fig. 4.

**Fig. 5:**
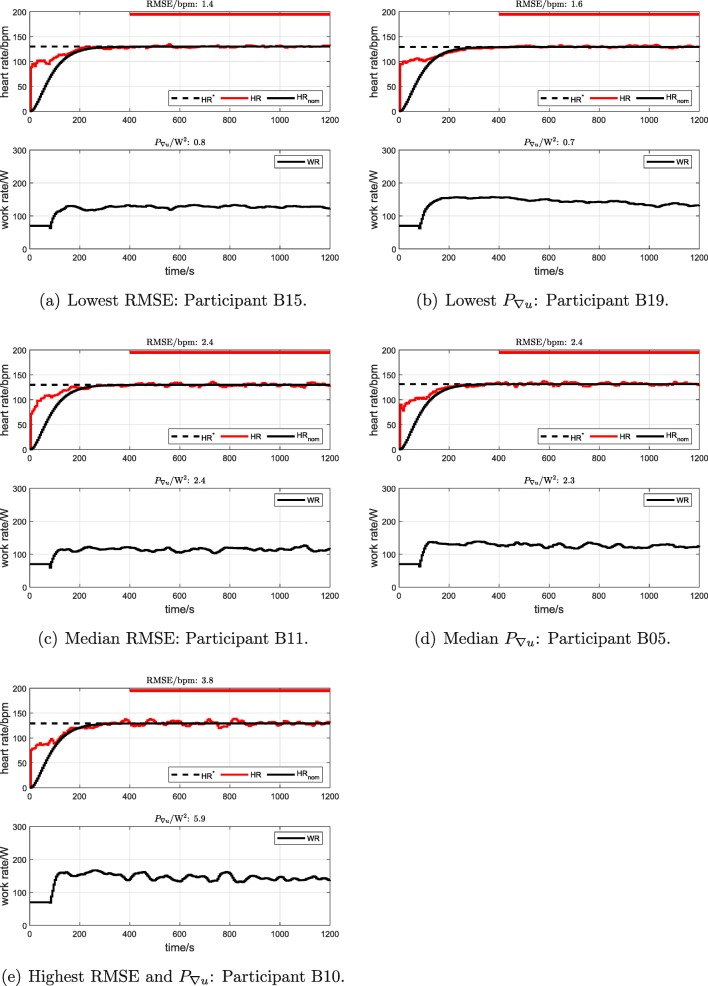
Participant cohort B: individual HR control tests with the minimum, median and maximum values of RMSE (parts a, c and e) and of *P*_∇*u*_ (parts b, d and e). “B15” refers to cohort B, participant number 15, etc. The red bars depict the outcome-evaluation interval from 400 to 1200 s

For participant cohort B (*n* = 24), cycling at a constant cadence of 70 rpm and tested with a constant HR target, RMSE was 2.46 bpm ± 0.59 bpm (range 1.39 bpm to 3.80 bpm) and *P*_∇*u*_ was 2.40 W^2^ ± 1.17 W^2^ (range 0.76 W^2^ to 5.93 W^2^). For participant cohort B (*n* = 24), cycling under the alternative condition of freely chosen cadence and, again, with a constant HR target, RMSE was 2.57 bpm ± 0.57 bpm (range 1.58 bpm to 3.40 bpm) and *P*_∇*u*_ was 2.39 W^2^ ± 1.02 W^2^ (range 0.69 W^2^ to 4.45 W^2^). Original data records for the individual cohort B tests with the minimum, median and maximum values of RMSE and of *P*_∇*u*_ are shown in Fig. 5 (note: minima, medians and maxima were obtained over all cohort B tests, i.e. including both the 70 rpm and the freely chosen cadence conditions).

The mean values of RMSE and *P*_∇*u*_ for the two cohort B conditions were found not to be significantly different: for RMSE, the *p* value was *p* = 0.45, and for *P*_∇*u*_, *p* = 0.96 (cadence of 70 rpm vs. freely chosen cadence, paired-samples two-sided *t* tests, significance level *α* = 0.05).

### Parametric plant model uncertainty

To facilitate analysis of performance and stability robustness properties of the feedback compensator *C*_fb_ in Eq. 4 (Section 3.3, below), a family of plant models of the form Eq. 1 was obtained in separate system identification studies. Three sets of models are considered here as follows:

1. Participant cohort A (*n* = 25; full study details are given in [22]): using a sample of 25 healthy males, the steady-state gain and time constant were estimated for exercise on the cycle ergometer to be *k* = 0.392 bpm/W ± 0.120 bpm/W (mean ± standard deviation; range 0.180 bpm/W to 0.796 bpm/W) and *τ* = 68.7 s ± 21.5 s (range 38.1 s to 120.2 s), respectively. In this study, participants were required to maintain a constant pedalling cadence of 70 rpm.
  (The nominal plant gain *k* = 0.392 bpm/W in Eq. 1 was taken as the mean value from this identification study. The nominal plant time constant *τ* = 65.6 s in Eq. 1 is the mean obtained across the 25 cycle ergometer values and 25 separate measurements with the same participants exercising on a treadmill, where *τ* = 62.5 s ± 18.5 s [range 34.3 to 110.1], see [22]).
2. Participant cohort B (*n* = 24): models for a separate sample of 24 healthy males were identified on the cycle ergometer using the same experimental protocol detailed in [22], with cadence = 70 rpm; this identification series is denoted B1. This resulted in the estimates *k* = 0.372 bpm/W ± 0.094 bpm/W (range 0.227 bpm/W to 0.565 bpm/W) and *τ* = 71.8 s ± 21.7 s (range 43.0 s to 133.2 s).
3. Participant cohort B (*n* = 24): this identification series, denoted B2, used the same cohort as in B1, but participants were allowed to freely choose their pedalling cadence. The outcomes were *k* = 0.364 bpm/W ± 0.074 bpm/W (range 0.197 bpm/W to 0.518 bpm/W) and *τ* = 65.7 s ± 24.2 s (range 26.5 s to 125.6 s).

**Fig. 6:**
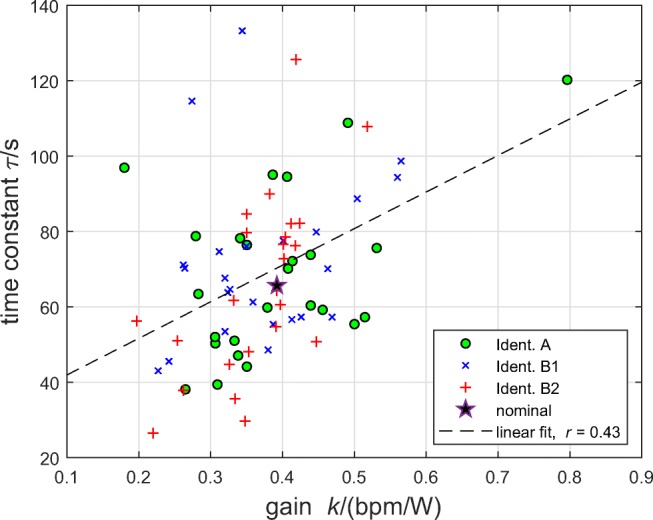
Dispersion of *k* and *τ* for 73 individual models from three system identification test series (Section 3.2). Ident. A: participant cohort A (*n* = 25, cadence 70 rpm). Ident. B1: participant cohort B (*n* = 24) with cadence 70 rpm. Ident. B2: participant cohort B (*n* = 24) with self-selected cadence. The star depicts the nominal model used for controller calculation (*k* = 0.392, *τ* = 65.6; Section 2.1). The dashed line is a linear fit (*r* = 0.43, *p* = 0.00017)

Dispersion of *k* and *τ* for the cycle ergometer for the three sets of models is displayed together with values for the nominal model in Fig. 6.

The mean values of *k* and *τ* obtained from identification series A, B1 and B2 are deemed consistent because statistical testing revealed no significant differences between different pairs of conditions: *τ*, B1 vs. B2 (*p* = 0.30); *k*, B1 vs. B2 (*p* = 0.65); *τ*, A vs. B1 (*p* = 0.62); *k*, A vs. B1 (*p* = 0.53); *τ*, A vs. B2 (*p* = 0.65); *k*, A vs. B2 (*p* = 0.33). For the B1 vs. B2 comparisons, paired-samples two-sided *t* tests were conducted; for A vs. B1/B2, independent-samples two-sided *t* tests were used.

For the series B1 and B2, there was no significant difference in mean RPE: 13.3 ± 1.4 vs. 13.1 ± 1.7, B1 vs. B2, *p* = 0.089 (paired-sample two-sided *t* test).

There was a positive linear correlation between *k* and *τ* (*r* = 0.43, *p* = 0.00017; Fig. 6).

### Robustness analysis

Performance and stability robustness properties of the feedback compensator *C*_fb_ in Eq. 4 were analysed using the family of 73 plant models obtained empirically as described above.

**Fig. 7:**
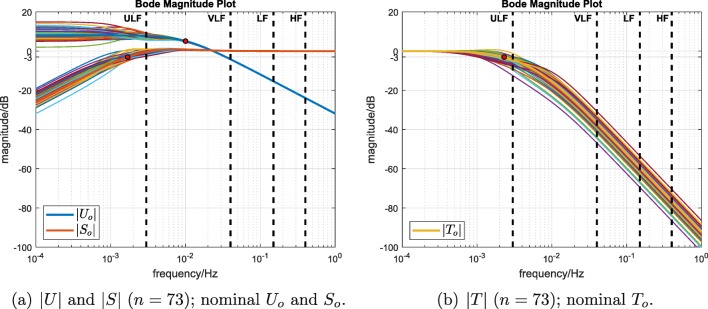
Performance robustness: sensitivity function magnitudes computed using the single feedback compensator *C*_fb_ together with the nominal plant *P*_*o*_ and the 73 identified plant models. The red dots mark the nominal − 3 dB bandwidths

Performance robustness was investigated by computing the closed-loop input-sensitivity, sensitivity and complementary sensitivity functions *U*, *S* and *T* (Eqs. 6–8) using the single feedback compensator *C*_fb_, Eq. 4, and the 73 plant models. The 73 magnitude plots |*U*|, |*S*| and |*T*| are displayed along with the nominal sensitivity functions (Bode magnitude plots, Fig. 7).

**Fig. 8:**
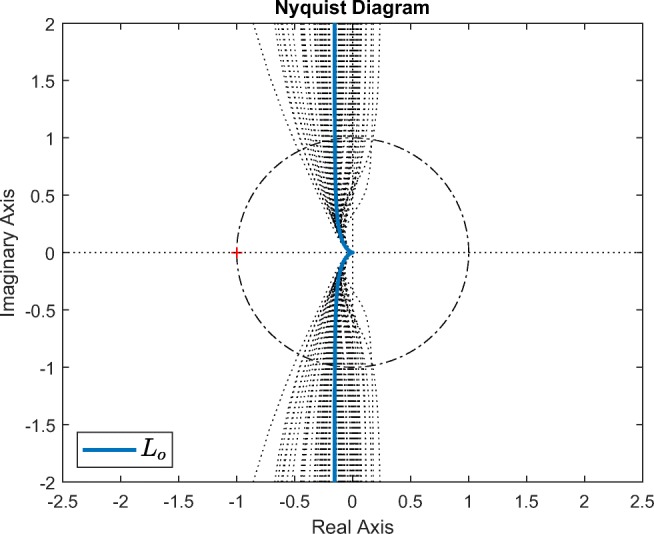
Stability robustness: Nyquist plots of nominal loop gain *L*_*o*_ = *C*_fb_*P*_*o*_ and of *L* = *C*_fb_*P* for the 73 instances of *P* (identified plant models). Gain margin is infinite for all models. Nominal phase margin is 81.2^∘^; minimal and maximal phase margins are 62.2^∘^ and 99.9^∘^, respectively

For investigation of stability robustness, the loop gain *L* (Eq. 5) was computed using the single *C*_fb_ transfer function together with the nominal plant *P*_*o*_ and the 73 identified plant models (Nyquist plots, Fig. 8). The nominal gain margin is infinite and the nominal phase margin is 81.2^∘^. The gain margin remains infinite across all 73 instances of *L*; the minimum phase margin is 62.2^∘^, which occurs for the model with *k* = 0.80 and *τ* = 120.2 (which lies at the upper-right corner of the *k*-*τ* plane, i.e. high values of both *k* and *τ* [Fig. 6]); the maximum phase margin is 99.9^∘^, which occurs for the model with *k* = 0.35 and *τ* = 29.7 (close to the lower-left corner of the *k*-*τ* plane, i.e. low values of both *k* and *τ* [Fig. 6]).

## Discussion

The single linear compensator was found to give highly accurate HR tracking performance in both experimental cohorts and under the different experimental conditions: mean RMSE was on the range 2.5 bpm to 3.1 bpm. Due to the dynamic nature of square-wave reference tracking, mean RMSE for this condition (3.1 bpm) was higher than for the two constant target regulation series (2.5 bpm and 2.6 bpm).

The input-sensitivity-shaping control design approach gives a simple, closed-form analytical procedure that allows the closed-loop bandwidth to be set in consideration of the broad-spectrum HRV disturbance. In the present set of experiments, this gave a stable and smooth control signal whose changes had low average power *P*_∇*u*_ (mean of 2.4 W^2^ for constant HR regulation and 10.3 W^2^ for square-wave tracking).

Although the HR response was represented using the approximation of a simple linear model of the form *y* = *P*_*o*_(*s*)*u* + *d* (Eq. 1, Fig. 1), where the term *d* represents the lumped effects of the HRV disturbance at a nominal operating point, it should be emphasised that human heart rate variability arises from complex interactions between the sympathetic and parasympathetic divisions of the autonomic nervous system [18]. These divisions are continuously engaged in regulation of cardiac output by adjustment of stroke volume and heart rate, thus leading to the observed variations in the time between individual beats. This HRV depends on many factors that are not dependent upon the control signal *u* (target work rate) including hydration level, ambient temperature and health status. Thus, it is not the purpose of the feedback control loop and, in particular, the control signal *u*, to directly influence the level of HRV. Rather, HRV is treated as a lumped, unmeasurable output disturbance *d*; the task of the controller is then, in the face of the unknown HRV disturbance *d*, to achieve a sufficient level of accuracy in the tracking of the target HR profile while maintaining an acceptable intensity of the control signal *u*. This amounts to the classical trade-off between tracking accuracy and control signal intensity: choice of a higher closed-loop bandwidth will tend to give a more dynamic controller resulting in lower tracking error but higher control signal intensity, and vice versa.

In comparison with the study of Kawada et al. [6], which employed a PI controller and evaluated only constant HR regulation, the RMSE values for regulation in the present work are slightly lower ($\sim $2.5 bpm here vs. 2.5 bpm to 3.8 bpm in [6]); but this comparison should be interpreted with caution since RMSE will also have been affected by the differing experimental conditions and the respective methods for controller-parameter tuning.

A direct comparison of the intensity of control signal activity between the two studies is not possible: here, this was evaluated using the average power of sample-to-sample changes in the control signal *P*_∇*u*_; but in [6], no quantitative assessment of control signal intensity was performed. It can be conjectured, however, that the control signal intensity when using a PI controller (as in [6]) would be higher. This is because, in the present work, the compensator, Eq. 2, was constrained at the outset to be strictly proper (low pass). This in turn gives a strictly proper, low-pass input-sensitivity function *U*_*o*_ because, from Eq. 6, *U*_*o*_ = *C*_fb_/(1 + *C*_fb_*P*_*o*_). Thus, when $\lim _{\omega \rightarrow \infty } |C_{\text {fb}}| = 0$, it follows that $\lim _{\omega \rightarrow \infty } |U_{o}| = \lim _{\omega \rightarrow \infty } |C_{\text {fb}}| = 0$. Thus, the control signal will not respond to disturbances at frequencies above the specified input-sensitivity bandwidth *p* (set here as frequency *f* = 0.01 Hz; see |*U*_*o*_| in Fig. 2).

In contrast, for a PI controller *C*_fb_(*s*) = *k*_*p*_ + *k*_*i*_/*s* with proportional gain *k*_*p*_ and integrator gain *k*_*i*_ (this is the exact structure employed in [6]), the magnitude of *C*_fb_ tends to the value *k*_*p*_ at high frequency. Consequently, |*U*_*o*_| also tends to the value *k*_*p*_ because, employing the condition that *P*_*o*_ is strictly proper (low pass), $\lim _{\omega \rightarrow \infty } |U_{o}| = \lim _{\omega \rightarrow \infty } |C_{\text {fb}}| = k_{p}$. This shows that, for a PI controller, the control signal will react to disturbance and noise inputs across the whole frequency spectrum.

Finally, in comparison with the study in [6], it is noted that the nominal plant gain used here for controller calculation (*k* = 0.39, mean from 25 participants) was very close to the value estimated in [6] (*k* = 0.42, mean from 10 participants). The nominal time constant used here cannot be compared because a non-parametric model was estimated in [6].

The performance of the controller proposed and tested in the present work can be compared with nonlinear strategies that have previously been applied to HR control. One nonlinear approach has been applied to HR control during both treadmill [4] and cycle ergometer [7] exercise. This nonlinear method is based upon a plant model where the control signal *u* appears in quadratic form, and where the controller cancels this term using the inverse nonlinearity, viz. the square-root function. This approach has the important theoretical property that global convergence of regulation errors is guaranteed for the class of nonlinear models considered. However, the experimental evidence provided in [4] and [7] is weak because no quantitative measures of controller performance were employed, and because short-duration tests were performed with only two (treadmill, [4]) or three (cycle, [7]) participants. Furthermore, a later independent study systematically compared this nonlinear approach to a linear PI controller using quantitative outcome measures and a cohort of 16 healthy male participants during treadmill exercise [26]. Using formal statistical analysis methods, this study found no significant difference between the linear and nonlinear controllers in HR tracking accuracy (for both controllers, RMSE was approximately 2.3 bpm) and in average control signal power. Moreover, the nonlinear controller was found to be highly sensitive at low control signal levels, which was attributed to the fact that the square-root function, which is included in the compensator, has a gain that tends to infinity as the control signal tends to zero.

The HR tracking accuracy reported in [26] for both the linear and nonlinear controllers, i.e. RMSE of approximately 2.3 bpm, is slightly lower than the range of 2.5 bpm to 3.1 bpm observed in the present work. This can likely be attributed to the non-strictly-proper nature of the linear/nonlinear controllers implemented in [26], in contrast to the strictly proper constraint applied here (Eq. 2): when the controller is not strictly proper, its gain does not roll off with frequency, thus making it more dynamic across the whole frequency range, which tends to drive down the RMSE; the price to be paid for this improved HR tracking accuracy, however, is an increased sensitivity to higher frequency HRV disturbances and consequent higher average control signal power.

Notwithstanding this critical analysis of nonlinear control strategies, further work is recommended to investigate appropriate nonlinear plant model and controller structures, while experimental evaluations are recommended that comprise quantitative performance-outcome measures and participant cohorts with sufficient sample size to allow formal statistical comparison with other linear/nonlinear approaches.

Within the present work, the quantification of parametric plant uncertainty showed that steady-state gains and time constants vary over a very wide range; overall, *k* was on the range 0.180 bpm/W to 0.796 bpm/W and *τ* ranged from 26.5 to 133.2 s (Section 3.2, Fig. 6). Despite this high level of plant dispersion, the controller was accurate and stable in all 73 experiments involving a total of 49 individual participants.

This empirically observed, high degree of controller robustness is underscored by the performance and stability robustness analysis (Section 3.3):

- Performance robustness: the magnitudes of the input-sensitivity and sensitivity functions, |*U*| and |*S*|, respectively, were found to be almost entirely unaffected by the plant variability at frequencies above the selected closed-loop bandwidth *p* (which corresponds to *f* = 0.01 Hz, Fig. 7a), i.e. in the frequency range that is important in relation to the behaviour of the control signal. Furthermore, the complementary sensitivity function magnitude |*T*| was found to be little affected at the lower end of the ultra low frequency band (Fig. 7b), i.e. at frequencies that are primarily important for reference tracking accuracy.
- Stability robustness: very large stability margins were evident across the whole family of plant models (Fig. 8): gain margin was infinite in all cases while the minimum phase margin remained large at 62.2^∘^ (nominal phase margin was 81.2^∘^).

## Conclusion

The single-linear, time-invariant controller was found to give accurate and stable performance with low values of the quantitative outcomes root-mean-square tracking error RMSE and average control signal power *P*_∇*u*_. The empirical evidence of controller robustness was corroborated by numerical analysis of key closed-loop transfer functions and stability margins across the available plant model family.

These results, taken together with data reported in Kawada et al. [6], demonstrate that highly accurate, stable and robust heart rate control can be achieved using LTI controllers of remarkably simple structure. Furthermore, the results highlight that design methods for HR control must give adequate attention to plant disturbances caused by physiological heart rate variability. The input-sensitivity approach evaluated in this work provides a direct and transparent method of addressing this challenge.

## Data Availability

The raw data supporting the conclusions of this manuscript will be made available by the authors, without undue reservation, to any qualified researcher.

## Glossary of terms

BMI: Body mass index
bpm: Beats/min
HR: Heart rate
HR^∗^: Target heart rate
HRmax: Maximal heart rate
HRmid: Mid-level heart rate
HRnom: Nominal (simulated) heart rate
HRMI: Heart rate monitor interface
HRV: Heart rate variability
*k*: Steady-state gain
LTI: Linear, time-invariant
*P*_∇*u*_: Average control signal power
PC: Personal computer
PI: Proportional-integral
PID: Proportional-integral-derivative
RMS: Root-mean-square
RMSE: RMS tracking error
RPE: Rating of perceived exertion
rpm: Revolutions per minute
*τ*: Time constant
USB: Universal serial bus
*v*: Speed
WR: Work rate
